# *Dendrobium officinale* Polysaccharides as a Natural Functional Component for Acetic-Acid-Induced Gastric Ulcers in Rats

**DOI:** 10.3390/molecules29040880

**Published:** 2024-02-16

**Authors:** Miao Zhang, Liba Xu, Long Chen, Huan Wu, Li Jia, Hua Zhu

**Affiliations:** 1 School of Chemistry and Chemical Engineering, Guangxi Minzu University, Nanning 530006, China; 17535764888@163.com (M.Z.); j13209211607@163.com (L.J.); 2 Guangxi Science Research Center of Traditional Chinese Medicine, Guangxi University of Chinese Medicine, Nanning 530200, China; xuliba15078772841@163.com (L.X.); tcmchenl1988@163.com (L.C.); 15278387621@163.com (H.W.); 3 Department of Analytical Chemistry and Food Science, Faculty of Food Science and Technology, University of Vigo, 36310 Vigo, Spain

**Keywords:** *Dendrobium officinale* polysaccharides, structure characterization, gastric ulcer, oxidative stress

## Abstract

*Dendrobium officinale* is an important edible and medicinal plant, with the *Dendrobium officinale* polysaccharide (DOP) being its primary active constituent, known for its diverse biological activities. In this study, DOP was extracted and characterized for its structural properties. The potential of DOP to ameliorate gastric ulcers (GUs) was investigated using an acetic-acid-induced GU model in rats. The results demonstrated that DOP exerted a multifaceted protective effect against GU, mitigating the deleterious impact on food intake and body weight in rats. DOP exhibited its protective action by attenuating cellular damage attributed to oxidative stress and inflammatory reactions mediated by enhanced activities of SOD, GSH, and GSH-PX, coupled with a downregulation in the expression of pro-inflammatory cytokines, including IL-1β, IL-6, and TNF-α. Furthermore, DOP effectively inhibited apoptosis in gastric mucosa cells of acetic-acid-induced GU rat models and facilitated the self-repair of damaged tissues. Remarkably, the DOP-200 and DOP-400 groups outperformed omeprazole in reducing the expression of IL-6 and malondialdehyde (MDA) in tissues, as well as IL-1β, IL-6, and TNF-α in serum. These groups also exhibited an improved expression of SOD in tissues and SOD, GSH, and GSH-PX in serum. A Western blot analysis of gastric mucosa demonstrated that the DOP-200 and DOP-400 groups significantly reduced the expression of NF-κBp65, phosphorylated NF-κBp65, FoxO3a, and Bim. The observed antagonism to GU appeared to be associated with the NF-κB cell pathway. Additionally, qRT-PCR results indicate that DOP reduced the mRNA transcription levels of IL-6, and TNF-α, which shows that the healing of GU is related to the reduction in the inflammatory reaction by DOP. However, the expression of EGF and VEGF decreased, suggesting that the mechanism of DOP inhibiting GU may not be directly related to EGF and VEGF, or there is an uncertain competitive relationship between them, so further research is needed.

## 1. Introduction

In both culinary and medicinal practices, *Dendrobium officinale* Kimura et Migo has a long history of use. Currently, *D. officinale* is frequently employed as a functional food to protect against gastric mucosal injuries [1]. In addition to minerals and amino acids, *D. officinale* also contains various bioactive compounds, such as flavonoids, phenols, and polysaccharides [2,3,4]. These components are known for their antioxidative, immune-enhancing, hepatoprotective, and stomach-strengthening properties [2,3]. The substantial presence of polysaccharides in *D. officinale* plays a pivotal role in its medicinal effects [5]. Previous research has established that the *Dendrobium officinale* polysaccharide (DOP) exhibits anti-inflammatory and antioxidant properties [6,7]. Plant polysaccharides have distinct characteristics, including large molecular weights and intricate structures, as well as their biological activity being directly or indirectly linked to their structure. To advance our understanding of *D. officinale* and its applications, further research is needed into the characteristics and biological activities of polysaccharides from *D. officinale*.

Gastric ulcers (GUs) represent a common benign peptic ulcer condition [8]. The causes of GU are diverse, with the clinical presentation encompassing epigastric pain, localized tissue inflammation in the gastric mucosa, heightened oxidative stress, and necrotic lesions, all of which significantly impair the quality of life [9,10,11]. Current evidence suggests that chronic peptic ulcers affect approximately 10% of the global population [12]. The main contributors to gastric ulcers are excessive gastric acid secretion, reduced protective mucus, and contact with gastric acid leading to damage to the stomach lining. Existing approaches for GU prevention and treatment primarily revolve around pharmaceutical interventions aimed at reducing or eliminating factors causing mucosal damage or enhancing the defense and repair mechanisms. Synthetic drugs remain the mainstay of treatment for GU [13], but they come with certain drawbacks, such as substantial side effects, susceptibility to drug resistance, and high costs [14]. In recent years, active natural ingredients have shown growing promise in the prevention and treatment of various human ailments [15]. Plant polysaccharides [16,17] have been found to possess anti-GU properties, underscoring the significance of investigating gastric mucosal damage. To date, the model of acetic-acid-induced gastric ulcers is closely related to human gastric ulcers, and our literature review revealed that gastric ulcers have not been reported to be protected by polysaccharides from *D. officinale*.

It is now understood that the initiation and progression of gastric ulcers is closely linked to oxidative stress and inflammation. The elimination of oxygen free radicals not only helps prevent gastric mucosal injuries but also facilitates the healing of existing injuries [18,19]. Key changes in oxidative stress markers such as superoxide dismutase (SOD), malondialdehyde (MDA), prostaglandin E-2 (PGE2), glutathione (GSH), and glutathione peroxidase (GSH-PX) play an important role in the development of GU. Additionally, GU can be considered as an inflammatory response to some extent. Proinflammatory cytokines like TNF-α, IL-6, and IL-1β can exacerbate GU injuries and promote gastric mucosal cell apoptosis, thereby exacerbating GU [20]. Consequently, the utilization of medications to reduce oxidative stress levels and proinflammatory factor expression can hinder the initiation or further progression of GU, as supported by numerous recent studies [21,22,23,24].

On the basis of the findings above, we hypothesized that utilizing DOP will mitigate oxidative stress and inflammation, inhibit apoptosis, and enhance the repair capacity of the gastric mucosa to effectively counter GU and achieve successful treatment of this patient population.

## 2. Results

### 2.1. Chemical Characteristics of DOP

**Figure 1:**
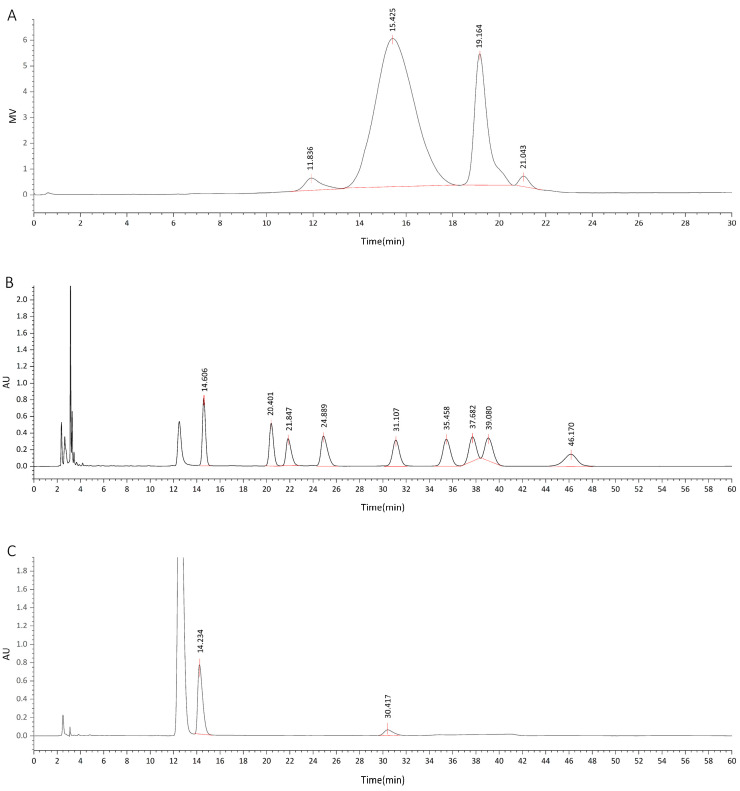
HPLC diagram of DOP. (**A**) shows the HP-GPC spectrum of DOP. (**B**) illustrates the mixed solution of monosaccharide standards, and (**C**) displays the HPLC monosaccharide composition diagram of DOP. In (**B**), the labels and peak times of nine monosaccharide standards are as follows: D-mannose (1, 14.606 min), rhamnose (2, 20.401 min), D-glucuronic acid (3, 21.847 min), D-galacturonic acid (4, 24.899 min), D-glucose (5, 31.107 min), galactose (6, 35.458 min), D-xylose (7, 37.682 min), L-(+)-arabinose (8, 39.08 min), and fucose (9, 46.17 min). In (**C**), the first peak (highest) was an interference peak. The peak time of the first red peak was 14.234 min, which closely matched the peak time of D-mannose. The peak time of the second red peak was 30.417 min, which was close to the peak time of D-glucose.

The determined total sugar content of DOP was 83.05%, with a moisture content of 13.77%, a total ash content of 0.99%, and a protein content of 0.00%. High-performance gel chromatography revealed a range of DOP molecular weights, varying from 2.27 × 10^6^ kDa to 3.7 × 10^2^ kDa to 0.46 kDa (as shown in Figure 1A). The results of HPLC identified the order of HPLC peaks in the mixed solution of nine monosaccharide standards, which were D-mannose, rhamnose, D-glucuronic acid, D-glucose, galactose, D-xylose, L-(+)-arabinose, fucose, and D-galacturonic acid (Figure 1B). By comparing this HPLC profile with that of DOP (Figure 1C), the monosaccharide composition of DOP was determined, and the molar percentage of the monosaccharide composition was calculated. Based on the results as shown in Table 1, DOP consisted primarily of mannose and glucose, with a molar ratio of 4.71:1.

**Figure 2:**
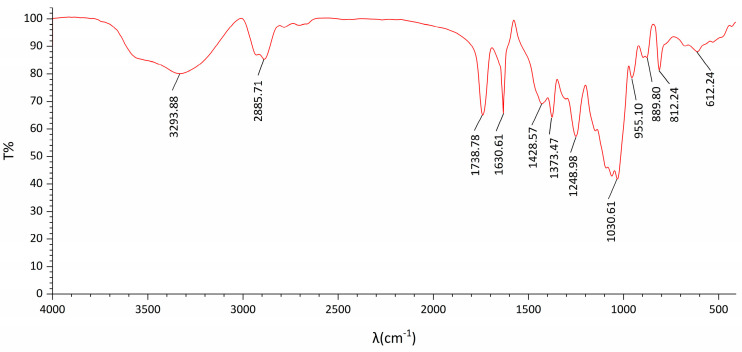
FI-IR of DOP. The absorption peak at 3293.88 cm^−1^ represents the hydrogen bond O−H stretching vibration, and the absorption peak at 2885.71 cm^−1^ represents C−H asymmetric stretching vibration, characteristic of sugar. Other notable absorption peaks were observed at 1738.73 cm^−1^ and 1630.61 cm^−1^ for the stretching vibration of the ester carbonyl group C=O and carboxyl carbonyl group C=O, respectively. Additionally, the absorption peak at 1738.78 cm^−1^ indicated the C−O stretching vibration of the acetyl group. Furthermore, the absorption peak at 1248.98 cm^−1^ indicated the stretching vibration of C−O in the ester group. A strong peak at 1030.61 cm^−1^ indicated the C−O−C stretching vibration of the glycoside structure, and an absorption peak at 955.10 cm^−1^ represented the stretching vibration of the pyran ring C−O. Additionally, the peaks at 889.80 cm^−1^ and 812.1 cm^−1^ were attributed to the absorption peaks of β−glucosidic bonds of glucose and mannose in the pyranose form.

**Figure 3:**
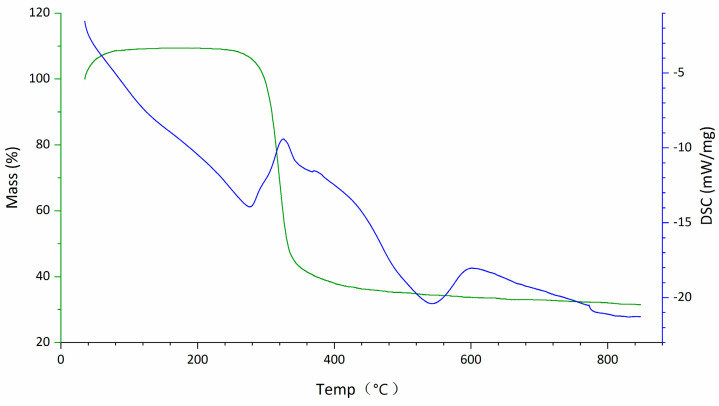
TG-DTA diagram of DOP. Between 37.94 °C and 239.4 °C, the TG curve displayed minimal variations, with a 0% mass loss, indicating that the polysaccharide mass remained stable. In this temperature range, the DSC curve exhibited a roughly linear downward trend, suggesting endothermic reaction of the polysaccharide as the temperature increased. From 239.4 °C to 351.98 °C, the TG curve showed a substantial decrease with a mass loss rate of 61.22%, signifying a significant change in the polysaccharide mass. The DSC curve displayed an inflection point and started to rise, indicating that the polysaccharide still exhibited endothermic behavior, albeit with a reduction in absorbed heat compared to the previous stage. Finally, from 351.98 °C to 848.00 °C, the TG curve changed gently again, with the mass loss rate stabilizing. The DSC curves continued to show a downward trend, confirming an ongoing endothermic reaction.

**Figure 4:**
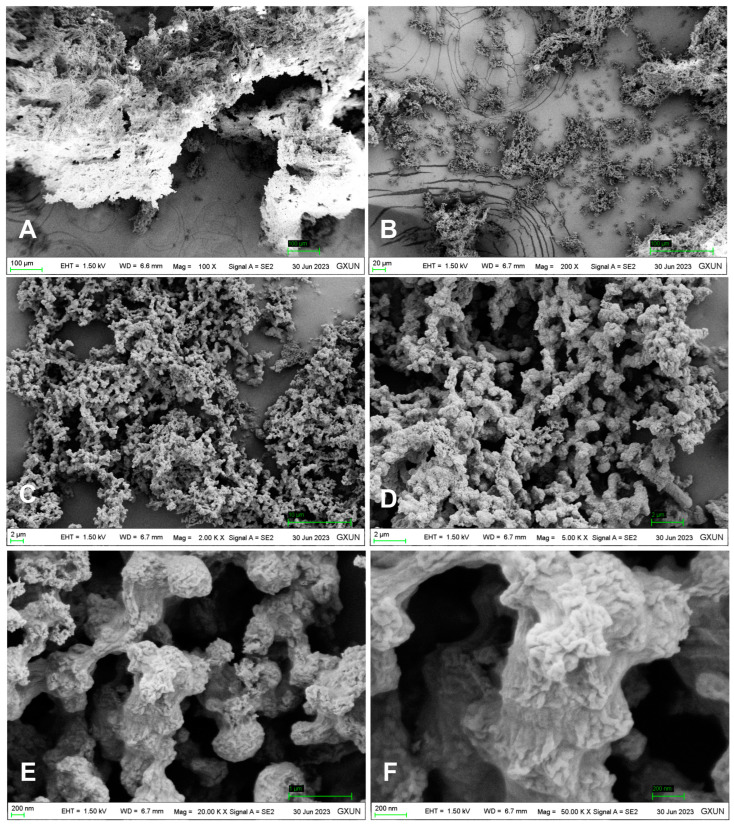
SEM image of the surface structure of DOP. SEM images of the DOP surface revealed distinct features. Images (**A**,**B**) display numerous particles of varying sizes on the DOP surface, while (**C**,**D**) show a clearer and more organized particle structure with close connections. Images (**E**,**F**) depict protruding particles on the DOP surface, forming columns.

The FT-IR spectrum of DOP (Figure 2) exhibited typical characteristic absorption peaks of polysaccharides. The TG-DTA diagram (Figure 3) indicated that the mass loss rate of DOP was nearly zero between 37.94 and 239.4 °C, signifying a stable structure. Subsequently, at temperatures ranging from 239.4 to 351.98 °C, the mass loss rate of DOP increased to 61.22%, suggesting the decomposition of the polysaccharide chemical bond. Between 351.98 and 848.00 °C, the mass loss rate of DOP gradually decreased and tended to be constant, and the polysaccharide was nearly completely decomposed, with the reaction being endothermic throughout the TGA-DTA experiment. SEM results showed that there were many particles of different sizes on the surface of DOP, which were clear in structure, closely linked, protruding from the surface, and interconnected in columns (Figure 4).

### 2.2. DOP Alleviated Decreased Feeding and Weight Loss Caused by GU

**Figure 5:**
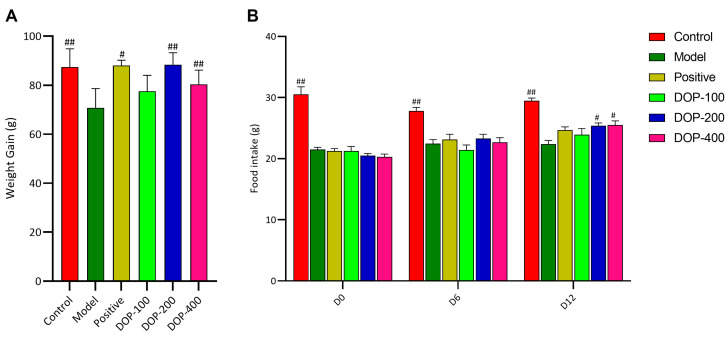
Effects of DOP on food intake and body weight in rats. (**A**) shows the weight gain of rats after treatment. (**B**) shows the changes of food intake of rats on the D0, D6 and D12 after drug administration. In the above figure, “#” indicates a significant difference compared with the model group (*p* < 0.05), and “##” indicates a very significant difference compared with the model group (*p* < 0.01).

A damaged gastric mucosa causes discomfort in the stomach during feeding, according to research, resulting in reduced food intake and weight loss in rats [25]. The changes in food intake and body weight among the different groups were investigated. Following drug intervention, the weight gain of rats in the control group, positive group, and DOP-200 and DOP-400 groups was significantly greater than that in the model group (Figure 5A). The results of food intake after drug administration showed that the food intake of rats in the control group was significantly higher than that in the model group (Figure 5B), indicating a notable reduction in food intake in rats due to acetic-acid-induced gastric ulcers. Moreover, as compared to the model group, the food intake of rats in the DOP-200 and DOP-400 groups showed a significant increase on the 13th day (D12), outperforming the positive group (Figure 5B).

### 2.3. Effects of DOP on Gastric Ulcers

**Figure 6:**
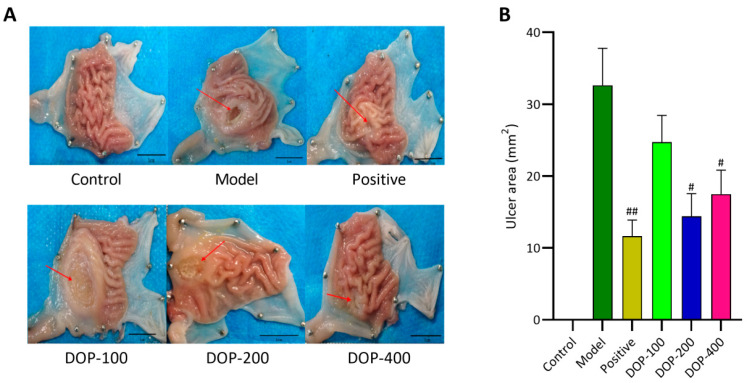
(**A**,**B**) Effects of DOP on gastric macroscopic changes in gastric ulcer rats induced by acetic acid. “#” indicates a significant difference compared with the model group (*p* < 0.05), and “##” indicates a very significant difference compared with the model group (*p* < 0.01).

The administration of acetic acid resulted in the development of significant gastric ulcers in the control groups where no therapeutic intervention for ulcer healing was administered. The control group did not exhibit significant mucosal defects in the gastric mucosa, and the gastric folds were clear. In contrast, the model group, DOP-100 group, and DOP-200 group exhibited varying degrees of gastric mucosal defects, forming oval or nearly round ulcer lesions. The folds in and around the gastric ulcer area were blurred or even disappeared. Significantly, the folds in and around the GU area had gradually recovered to some extent in the positive, DOP-200, and DOP-400 groups (Figure 6A). The area of GU serves as one of the most intuitive indices for evaluating the anti-gastric-ulcer effect of drugs. According to Figure 6B, it was found that the GU area of the model group was significantly greater than that of the control group (*p* < 0.01). After drug intervention, the area of GU decreased in the positive and DOP groups, and a significant difference was not found between the DOP-100 and model groups (*p* > 0.05). The other groups were able to significantly reduce the GU area, especially the positive group.

### 2.4. Histopathological Evaluation of Gastric Ulcer

**Figure 7:**
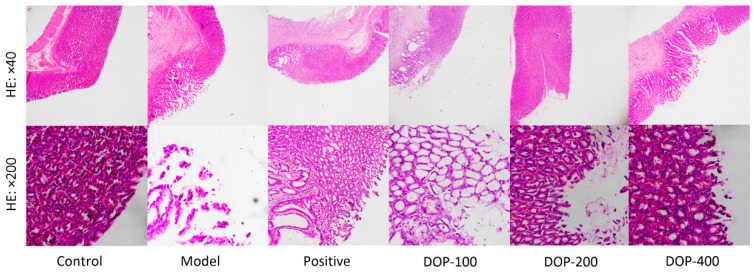
Effects of DOP on gastric pathological changes in gastric ulcer rats induced by acetic acid.

Significant damage to the gastric mucosa in rats was observed with HE staining (Figure 7). Gastric mucosa cells in the control group were neatly arranged with no evidence of damage or inflammation. Conversely, the model group exhibited obvious structural damage to gastric mucosal tissue, with ulcer injury extending to the muscular layer and noticeable lymphocyte infiltration. The mucosal morphology around the ulcer in the positive group tended to normalize, with a small amount of lymphocyte infiltration. In the DOP-100 group, the gastric mucosa displayed defects, evident structural disruption, and a substantial number of infiltrated lymphocytes. The morphological structure of the gastric mucosa in the DOP-200 and DOP-400 groups tended to normalize, but a small amount of lymphocyte infiltration was still observed in the gastric mucosa of the DOP-200 group.

### 2.5. DOP Inhibited Gastric Mucosal Cell Apoptosis

**Figure 8:**
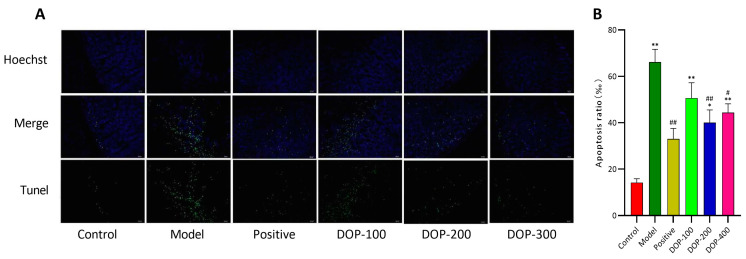
Effects of DOP on DNA damage of gastric mucosal cells in gastric ulcer rats induced by acetic acid (TUNEL staining, 400×). (**A**) is a fluorescent staining diagram. (**B**) is the graph of apoptosis ratio. “#” indicates a significant difference compared with the model group (*p* < 0.05), and “##” indicates a very significant difference compared with the model group (*p* < 0.01); “*” indicates a significant difference compared with the control group (*p* < 0.05), and “**” indicates a very significant difference compared with the control group (*p* < 0.01).

This study used TUNEL staining to assess apoptotic cells in the gastric tissues. The nuclei of normal cells appeared to be weakly fluorescent and blue, while the nuclei of apoptotic cells were brightly stained green. In the control group (Figure 8A), the results showed that the cells of gastric tissue were arranged closely and regularly, with predominantly blue-stained nuclei and only sporadic bright green spots, indicating a very small area and a low number of apoptotic cells. In the model group, the arrangement of gastric tissue cells was loose and disorganized, with an increase in the number of points displaying bright-green-stained nuclei and a larger area, signifying a significant increase in the rate of acetic-acid-induced apoptosis in gastric tissue cells compared to the control group. Following drug interventions, the arrangement of gastric tissue cells in the positive, DOP-200, and DOP-400 groups returned to a close and regular arrangement, with most of the nuclei staining blue and a significant reduction in bright green spots. Notably, as shown in Figure 8B, both the DOP-200 and DOP-400 groups significantly reduced apoptosis (*p* < 0.05), with the DOP-200 group demonstrating the most significant inhibition of apoptosis (*p* < 0.01).

### 2.6. DOP Regulated the Expression Levels of Inflammatory Cytokines in Gastric Tissue and Serum

Oxidative stress and inflammation are important triggers of gastric ulcers [26]. Proinflammatory factors such as IL-1β, IL-6, TNF-α, and PEG2 play a crucial role in the inflammatory response [27]. TNF-α stimulates IL-1β production and cell apoptosis, leading to delayed ulcer healing [28]. IL-6 stimulates immune cells to produce enzymes that damage ulcer tissue [29]. Therefore, reducing levels of TNF-α, IL-1β, and IL-6 may promote ulcer healing. The decrease in PGEs indicates a mucosal ulcer [30], and PGE2 helps with gastric mucus secretion and speeds up ulcer healing [31]. So, high levels of PGE2 are good for healing gastric ulcers. This study examines how DOP protects against gastric ulcers by looking at IL-1, IL-1β, TNF-α, and PGE2 levels.

**Figure 9:**
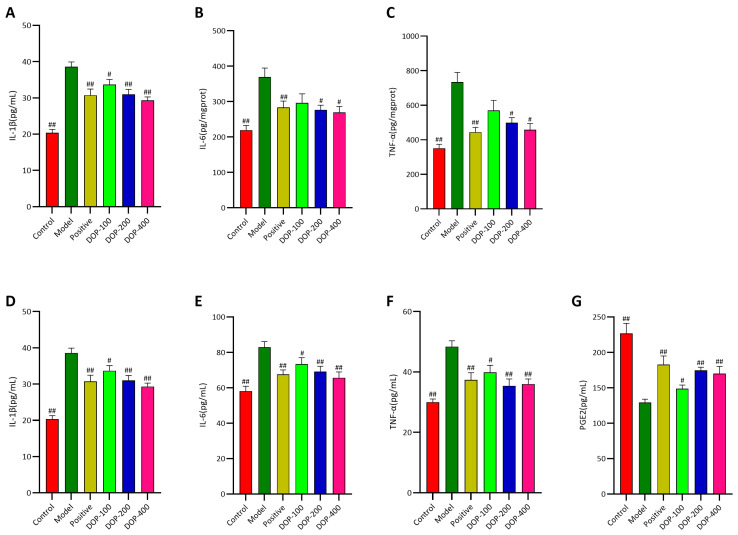
Effects of DOP on inflammation in gastric tissue and serum. (**A**–**C**) indicate the activities of IL-1β, IL-6, and TNF-α in rat stomach tissue, respectively. (**D**–**G**) indicate the contents of IL-1β, IL-6, TNF-α, and PEG2 in rat serum, respectively. In the above figure, “#” indicates a significant difference compared with the model group (*p* < 0.05), and “##” indicates a very significant difference compared with the model group (*p* < 0.01).

As illustrated in Figure 9A–C, compared with the control group, the expression levels of IL-1β, IL-6, and TNF-α in the gastric tissues of rats in the model group were significantly higher (*p* < 0.01), suggesting that the increased levels of inflammatory cytokines (IL-1β, IL-6, and TNF-α) were associated with gastric ulcers. Upon the administration of drug interventions, there was a notable reduction in the levels of IL-1β, IL-6, and TNF-α in the gastric tissues of the positive group compared to the model group (*p* < 0.01), indicating that the treatment of gastric ulcers with the drug was associated with a decrease in the levels of these inflammatory cytokines. The results confirmed that DOP was effective in reducing the levels of inflammatory factors (IL-1β, IL-6, and TNF-α) in the gastric tissues of rats with gastric ulcers, with the most significant effects observed in the DOP-200 and DOP-400 groups (*p* < 0.05 or *p* < 0.01).

As shown in Figure 9D–F, the trend of serum levels of IL-1β, IL-6, and TNF-α in all groups mirrored that of the gastric tissue groups, with a decrease in the drug intervention groups and an increase in the model group. The serum levels of IL-1β, IL-6, and TNF-α were significantly higher in the model group compared to the control group (*p* < 0.01), while PGE2 was significantly decreased (*p* < 0.01). The serum levels of IL-1β, IL-6, and TNF-α were significantly reduced in the positive group compared to the model group (*p* < 0.01), while PGE2 was significantly increased (*p* < 0.01). Notably, the effects of DOP were comparable to those of the positive group. The expression levels of IL-1β, IL-6, and TNF-α in the serum of rats in all DOP groups were significantly reduced (*p* < 0.05 or *p* < 0.01), and PGE2 was significantly increased (*p* < 0.05 or *p* < 0.01). Moreover, the efficacy of the DOP-200 and DOP-400 groups was superior to the positive group.

### 2.7. DOP Regulated the Expression Levels of Oxidative Stress Factors in Gastric Tissue and Serum

**Figure 10:**
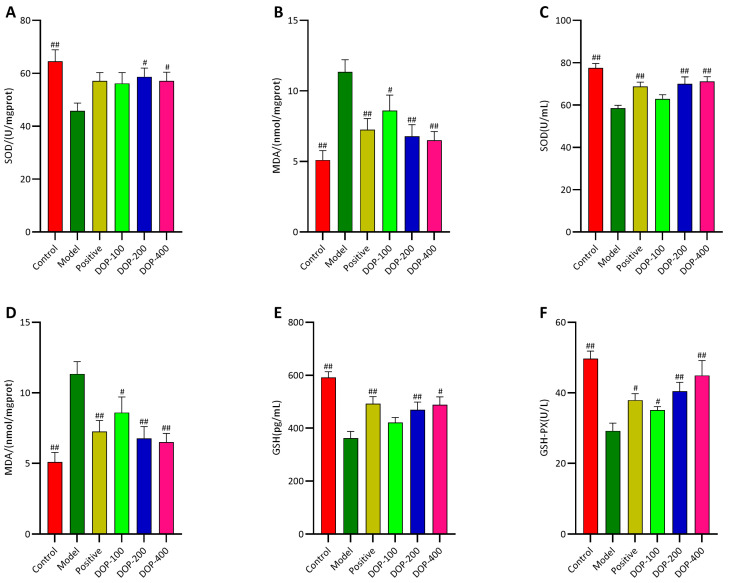
Effects of DOP on oxidative stress in gastric tissue and serum. (**A**,**B**) indicate the levels of SOD and MDA in rat serum, respectively. (**C**–**F**) indicate the activities of MDA, SOD, GSH, and GSH-PX in rat serum, respectively. In the above figure, “#” indicates a significant difference compared with the model group (*p* < 0.05), and “##” indicates a very significant difference compared with the model group (*p* < 0.01).

As depicted in Figure 10A,B, the levels of SOD in the gastric tissues of the model group were significantly lower (*p* < 0.01), and MDA levels were significantly higher as well (*p* < 0.01) compared to the control group. In contrast, compared with the model group, the levels of SOD in the gastric tissues of the positive group increased, and the levels of MDA significantly decreased (*p* < 0.01). In both the DOP-200 and DOP-400 groups, SOD levels in the gastric tissues were significantly increased (*p* < 0.05), and MDA levels were significantly reduced (*p* < 0.05 or *p* < 0.01). Notably, the DOP-200 and DOP-400 groups exhibited superior effects in reducing MDA expression compared to the positive group.

In serum, compared with the control group, the levels of SOD, GSH, and GSH-PX in the model group were significantly decreased (*p* < 0.01), and MDA was significantly increased (*p* < 0.01) (Figure 10C–F). However, in comparison to the model group, the levels of SOD, GSH, and GSH-PX in the positive group significantly increased (*p* < 0.05 or *p* < 0.01), and the level of MDA decreased significantly (*p* < 0.05). The expression levels of SOD, GSH, GSH-PX, and MDA in all DOP groups followed the same trends as those in the positive group. The expression levels of SOD, GSH, and MDA in the DOP-100 group showed no significant difference (*p* > 0.05), but significant differences (*p* < 0.05) or highly significant differences (*p* < 0.01) were observed in the other DOP groups.

### 2.8. DOP Inhibited mRNA Transcription of EGF, VEGF, IL-6, and TNF-α

**Figure 11:**
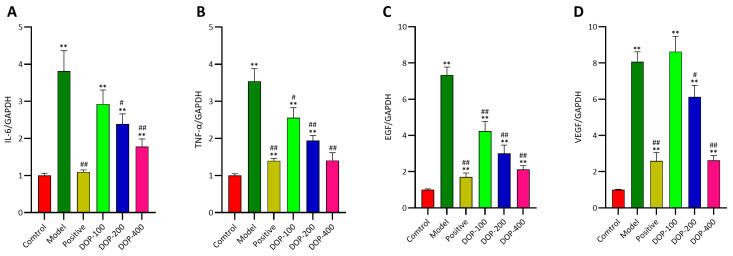
(**A**–**D**) The EGF, VEGF, IL-6, and TNF-α mRNA expression levels in gastric tissues were investigated by RT-qPCR. “#” indicates a significant difference compared with the model group (*p* < 0.05), and “##” indicates a very significant difference compared with the model group (*p* < 0.01); “**” indicates a very significant difference compared with the control group (*p* < 0.01).

It has been established that EGF, VEGF, IL-6, and TNF-α are crucial factors in the development of gastric ulcers, and their levels are associated with the healing or deterioration of these ulcers. As shown in Figure 11, mRNA expression levels of EGF, VEGF, IL-6, and TNF-α in gastric tissues in all groups were determined using RT-qPCR. The experimental results revealed that the mRNA expression levels of EGF, VEGF, IL-6, and TNF-α in the model group were observably higher than those in the control group (*p* < 0.01). In comparison to the model group, the mRNA expressions of EGF, IL-6, TNF-α, and VEGF in all drug intervention groups were significantly reduced, except for the DOP-100 group (*p* < 0.05). In particular, the mRNA expression of IL-6 and TNF-α in the DOP-200 and DOP-400 groups was significantly decreased, indicating that these groups could effectively inhibit the further deterioration of gastric ulcers. Both the positive group and DOP groups exhibited a reduced mRNA expression of EGF, which can facilitate ulcer healing. Similarly, the positive group and DOP-200 and DOP-400 groups significantly reduced the mRNA expression of VEGF, which is also beneficial for ulcer healing. Nonetheless, further research is warranted to fully understand this phenomenon.

### 2.9. DOP Downregulated the Expression of NF-κBp65, P-NF-κBp65, FoxO3a, and Bim in Gastric Mucosa

**Figure 12:**
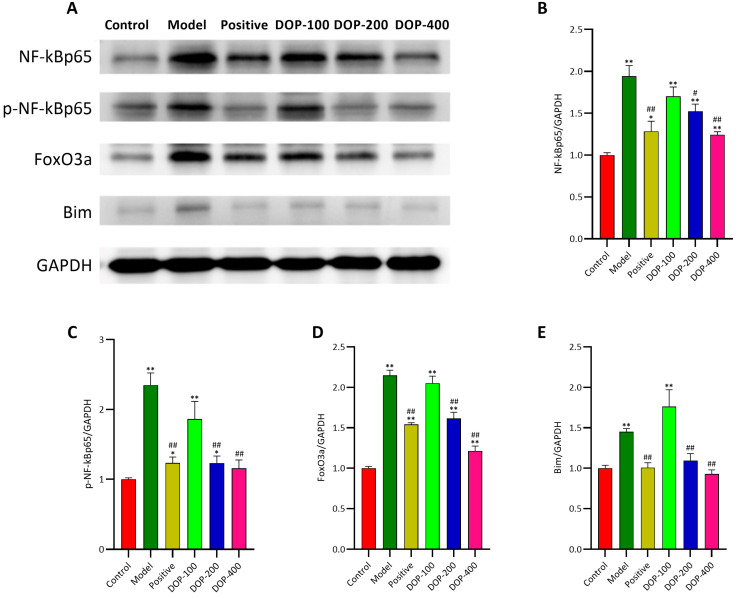
Effects of DOP on the expression of NF-κBp65, p-NF-κBp65, FoxO3a, and Bim proteins. (**A**) is the development diagram. The Western blotting analysis of the protein expression of (**B**) NF-κBp65, (**C**) p-NF-κBp65, (**D**) FoxO3a, and (**E**) Bim gastric tissues. “#” indicates a significant difference compared with the model group (*p* < 0.05), and “##” indicates a very significant difference compared with the model group (*p* < 0.01); “*” indicates a significant difference compared with the control group (*p* < 0.05), and “**” indicates a very significant difference compared with the control group (*p* < 0.01).

NF-κBp65, P-NF-κBp65, FoxO3a, and Bim are key factors related to cell inflammation and apoptosis. As demonstrated in Figure 12, Western blotting revealed that the expression levels of NF-κBp65, P-NF-κBp65, FoxO3a, and Bim proteins in the mucosa surrounding gastric ulcers in the model group were significantly higher than in the control group (*p* < 0.01). In comparison to the model group, the expression levels of NF-κBp65, P-NF-κBp65, FoxO3a, and Bim proteins in the positive group and the DOP-200 and DOP-400 groups were significantly decreased.

## 3. Materials and Methods

### 3.1. Chemicals and Reagents

Plants were collected from Nanning, China, and verified by Professor Jianbei Teng from the Department of the key laboratory of Zhuang and Yao ethnic medicine at Guangxi University of Chinese Medicine. Omeprazole enteric-coated capsules (Yuekang Pharmaceutical Group Co., Ltd., Beijing, China), a BCA protein assay kit (Biosharp, Hefei, China), and kits for the quantification of superoxide dismutase (SOD), malondialdehyde (MDA), interleukin-1β (IL-1β), and interleukin-6 (IL-6) (Jiancheng Bioengineering Institute of Nanjing, Nanjing, China) were used. Kits for tumor necrosis factor-ɑ (TNF-ɑ), reduced glutathione (GSH), glutathione peroxidase (GSH-PX), and prostaglandin E-2 (PGE2) (Jiangsu Enzyme Immunity Industry Co., Ltd., Jiangsu, China) were used. Antibodies against NF-κBp65, FoxO3a, and GAPDH (Proteintech Group, Chicago, IL, USA) and antibodies against P-NF-κBp65 and Bim (Abcam, Shanghai, China) were used. Horseradish-peroxidase-conjugated goat anti-rabbit and horseradish-peroxidase-conjugated goat anti-mouse secondary antibodies (Beyotime Biotechnology Company, Shanghai, China), a total RNA extraction kit (TRIzol method) (Shanghai Yuduo Biotechnology Co., Ltd., Shanghai, China), an SYBR Green PCR kit and a reverse transcription kit (Thermo Fisher Scientific, Waltham, MA, USA), a Tunel assay kit (Beyotime Biotechnology Company, Shanghai, China), DEPC (Beyotime Biotechnology Company, Shanghai, China), a DAB chromogenic kit (Beijing Zhongshan Golden Bridge Biotechnology Co., Ltd., Beijing, China), D-anhydrous glucose, rhamnose, D-glucuronic acid, D-galacturonic acid, galactose, D-xylose, L-(+)-arabinose, fucose (the Institutes for Food and Drug Control, Beijing, China), and other reagents were purchased from reagent companies.

### 3.2. Polysaccharide Preparation and Chemical Characterization

**Figure 13:**
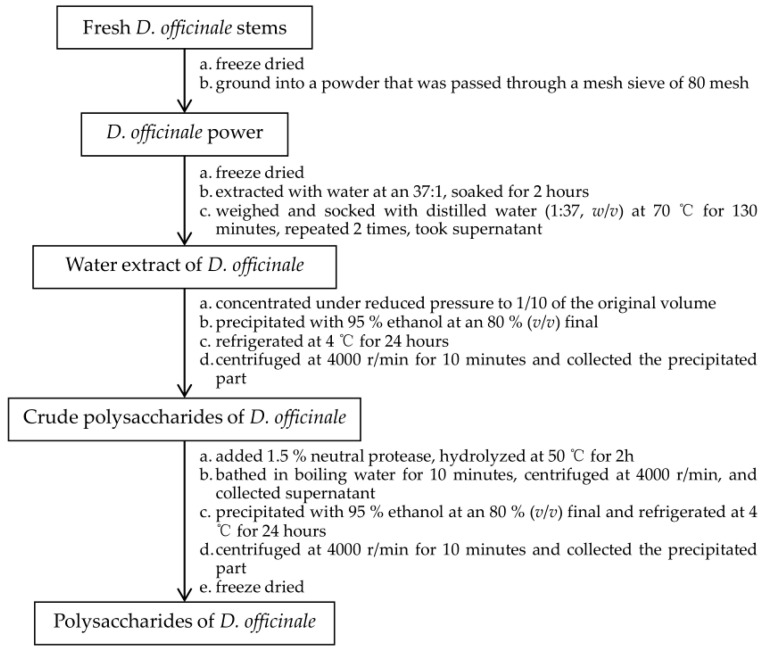
The detailed extraction method of DOP.

The DOPs were extracted by water extraction and alcohol precipitation [32] and the protein was removed by the neutral protease method. The detailed extraction method of DOP is summarized in Figure 13.

The total sugar content of DOP was determined using sulfuric acid phenol colorimetry [2]. In brief, 1 mL of the DOP solution was taken and mixed with 1 mL of a 5% phenol solution, and the mixture was then quickly added to 5 mL of concentrated sulfuric acid and subjected to a boiling water bath for 20 min, followed by an ice water bath for 10 min. The absorbance of the samples was measured at 485 nm by an ultraviolet and visible spectrophotometer (UV) (UV-2600, Shimadzu, Japan). The content of the DOP polysaccharide was calculated using D-glucose as a reference substance. Protein content was determined by the Coomassie brilliant blue method [33], and we measured 1 mL of the DOP solution, added 5 mL of the Coomassie brilliant blue solution, measured the absorbance at 595 nm by UV, and calculated the content of protein in DOP with bovine serum albumin as the standard. Total ash content was determined using the combustion method [33], following these steps: the DOP was burned at 550 °C for 3 h, weighed, re-burned, and weighed again until a constant weight was achieved. The total ash content was then calculated based on the residue weight. Moisture content was determined using the loss-on-drying method [33], which involved drying the sample at 105 °C for 5 h, weighing it, baking it again, and weighing it until a constant weight was obtained. The total moisture content was then calculated based on the weight lost. 

The monosaccharide composition was determined according to the method of Zhao et al. [34], with modifications. Briefly, acid hydrolysis was conducted using trifluoroacetic acid: we added a trifluoroacetic acid solution to the polysaccharide solution and hydrolyzed at 120 °C for 2 h, then added methanol and blow-dried by nitrogen, and repeated this three times. This was followed by 1-phenyl-3-methyl-5-pyrazolone (PMP) derivatization, with the below steps: A solution of NaOH was introduced into the hydrolyzed sample in order to achieve a neutral pH. Subsequently, a solution of PMP methanol was added to initiate a reaction at a temperature of 70 °C for a duration of 100 min. HCl was then employed to neutralize the reaction, followed by blow-drying by nitrogen. Ultrapure water was subsequently introduced for dissolution purposes, while chloroform was added thrice for extraction. The aqueous phase was retained and determination took place using HPLC. Nine monosaccharides, D-glucose, D-mannose, D-glucuronic acid, D-xylose, galactose, fucose, L-(+)-arabinose, rhamnose, and D-galacturonic acid, were derivatized with PMP. The HPLC detection conditions were as follows: a phosphate buffer (pH 6.7) and acetonitrile were used as mobile phase eluents, and the mobile phase solution ratio was 83:17. The chromatographic column was a ZORBAX SB-C18 column (4.6 mm × 250 mm, 5 µL, Agilent, Santa Clara, CA, USA), and a photo-diode array (PDA) detector (2998 PDA Detector, waters) was used with a detection sample volume of 10 µL, a detection flow rate of 1.0 mL/min, and a detection wavelength of 245 nm. Dextran with different molecular weights (800, 5000, 10,000, 50,000, and 1,000,000 D) served as standards. High-performance gel permeation chromatography (HP-GPC) (Waters e2695, IR Detector, PL aquagel-OH MIXED-H chromatographic column) was used to determine the molecular weight of DOP (5 mg/mL), with a sample volume of 20 µL, a column temperature of 35 °C, and a flow rate of 0.5 mL. DOP’s structural characteristics were determined by FT-IR (Spectrum TWO, PerkInElmer, Shelton, CT, USA) and a TG-DSC analyzer (STA 449 C, NETZSCH-Gerätebau GmbH, Wittelsbacherstrasse, Germany).

### 3.3. Animal Experiments and Design

Six-week-old male SD rats (Certificate No. SCXK (Xiang) 2018-0002) were procured from Hunan SJA Laboratory Animal Co., Ltd. (Hunan, China) and were housed in the controlled environment of the SPF experimental animal center at Guangxi University of Chinese Medicine, where the room temperature was kept at 20.0 °C to 25.0 °C, and relative humidity was between 40.0% and 70.0%. Prior to the formal experiment, rats were provided with food and water ad libitum and allowed to adapt to the environment for one week. This study’s animal experiment protocol was approved by the Animal Care and Use Ethics Committee at Guangxi University of Chinese Medicine (Certificate number: DW20211205-302), and all animal experiments were conducted according to Chinese and international animal welfare and ethics standards.

This study employed a model of acetic acid inducing chronic gastric ulcers in rats, modified from Okabe and Pfeiffer’s method [35]. After a 24 h fast, rats were subjected to laparotomy under anesthesia, and a solution of 20% acetic acid was injected into the serosa layer of the stomach in the amount of 0.1 mL at a point approximately 0.5 cm from the upper part of the pylorus. Sutures were then placed in the abdominal wall.

After a one-week acclimation period, we randomly divided 60 healthy rats into six groups: the control group, model group, positive group, DOP-100 group, DOP-200 group, and DOP-400 group. Except for the control group, in which rats underwent surgery but were not injected with acetic acid, all other groups of rats were modelled with acetic-acid-induced gastric ulcers. Starting from the day following the surgery, control and model group rats received pure water by gavage. Rats in the positive group were administered omeprazole (4.2 mg/kg) by gavage, while rats in the DOP-100, DOP-200, and DOP-400 dose groups were given DOP at doses of 100 mg/kg, 200 mg/kg, and 400 mg/kg, respectively, with a dosage volume of 10 mL/kg. This administration regimen was followed once daily for 14 days. Following the final administration, rats fasted for 14 to 16 h, were anesthetized, and blood was collected from the abdominal aorta. The blood samples were centrifuged for 10 min at 3000 r/min after standing for 2 h, and the supernatant was stored at −80 °C. Subsequently, the rats were euthanized by acute blood loss, the stomach was quickly isolated, and the gastric cardia and pylorus were ligated. Following the cutting of the stomach, a normal saline rinse was given, and the stomach was removed, and fixed with pins for observation and photography. Gastric tissue with an area of approximately 1 cm^2^, selected around the ulcer lesion (the same position on the stomach without ulcers), was divided into two parts along the longest diameter center of the ulcer. One part was fixed in 10% neutral formalin, while the other part was weighed immediately and stored at −80 °C.

### 3.4. Food Intake and Weight

Rat food intake and weight were assessed as indicators of gastric ulcer severity. In this study, rat food intake and weight were measured during the experiment. (1) Measurement of food intake: Starting from Day 0, food intake for rats in each cage was measured on Days 0, 6, and 12. (2) Weight determination: Weights were recorded at the start of administration (Day 0) and subsequently on Days 3, 6, 9, and 12.

### 3.5. Determination of Ulcer Inhibition Rate

Upon complete removal of the stomach, it was flattened on a white background board (white foam board), cut along the stomach’s greater curvature, and secured. A vernier caliper was used to measure the longest and shortest diameters of the ulcer, and the ulcer area (mm^2^) was calculated (ulcer area = π × longest diameter × shortest diameter × 1/4). Each sample was scored for a gastric ulcer according to predefined scoring criteria (healing score 0, superficial mucosal erosion score 1, deep ulcer or transmural necrosis score 2, perforation or penetrating ulcer score 3). The inhibition rate (%) of each drug on ulcers was calculated using the following formula:

(1) $$R=\frac{A_{M}-A_{D}}{A_{M}}\times 100\%$$

Note:

*R*: ulcer inhibitory rate.

*A_M_*: average ulcer area of model group.

*A_D_*: average ulcer area of drug treatment group.

### 3.6. Histopathological Evaluation and Gastric Mucosal Cell Apoptosis Assay

After the stomach was fully excised, the gastric mucosa was fixed in neutral formalin at 10% for 24 h. Subsequently, it was dehydrated, embedded in paraffin, sectioned, stained with hematoxylin–eosin, and observed under a microscope to assess the pathological changes in the gastric mucosa. TUNEL was used to determine whether gastric mucosal cells were undergoing apoptosis. There was weak fluorescence in normal nuclei and strong green fluorescence in apoptotic nuclei. After staining, gastric mucosal tissue slices were analyzed under a fluorescence microscope. The fields of view were selected for five or more consecutive times, and the number of apoptotic cells observed was recorded. The apoptotic rate of gastric mucosal cells was then calculated as follows: apoptotic rate = number of apoptotic cells/total number of counted cells × 100%.

### 3.7. Biochemical Indicator Assay

#### 3.7.1. Determination of Oxidative Stress Factors and Inflammatory Factors in Gastric Tissue

Gastric tissue was weighed and chopped, and ice-cold physiological saline was added at a weight-to-volume ratio of 1:9. A homogenate of 10% gastric tissue was prepared using a homogenizer. Subsequently, the homogenate of gastric tissue was centrifuged at 3000 r/min for 10 min, and the supernatant was collected. Oxidative stress factors SOD and MDA expression levels were measured using a kit, and the expression levels of IL-1β (pg/mL), IL-6 (pg/mL), and TNF-α (pg/mL) were determined using ELISA (Helsinki, Finland).

#### 3.7.2. Determination of Inflammatory Cytokines and Oxidative Stress Factors in Serum

After anesthesia in rats, the abdominal aorta was sampled for blood. The whole blood was left standing for 2 h, then 3000 r/min centrifugation for 10 min was performed, and the supernatant was collected. The expression levels of oxidative stress factors, such as MDA, SOD, GSH, and GSH-PX, in serum were measured using the corresponding kits. ELISA was used to determine the levels of inflammatory factors, such as PGE2, IL-1β, IL-6, and TNF-α, in terms of their expression.

### 3.8. Quantitative Real-Time PCR

Gastric tissues from each group were weighed and thoroughly ground in liquid nitrogen, and RNA was extracted following the protocol provided by the RNA extraction kit. Subsequently, the reverse transcription kit was used to synthesize cDNA. The reaction mixture consisted of 4 µL of a 5× reverse transcription buffer, 1 µL of an RNase inhibitor, 1 µL of a downstream universal primer, 1 µL of dNTPs, 1 µL of reverse transcriptase MMLV, 8 µL of DEPC-treated water, and 4µL of an RNA template, for a total volume of 20 µL. The reaction conditions were as follows: 42 °C for 40 min, followed by 85 °C for 5 min to inactivate MMLV. Following this, cDNA was subjected to PCR amplification. The amplification mixture consisted of 10 µL of SYBR Green Mix, 0.4 µL of upstream primer F, 0.4 µL of downstream primer R, 7.2 µL of ddH_2_O, and 2 µL of a cDNA template, resulting in a total volume of 20 µL. Amplification conditions included an initial denaturation for 10 min at 94 °C; afterwards, denaturation was performed for 20 s at 94 °C for 40 cycles, followed by annealing at 55 °C for 20 s, and 20 s of extension at 72 °C. Data were collected using the instrument’s proprietary software (ABI Prism 7500 SDS software Version 2.4), with an emphasis on detecting mRNA expression levels of EGF, VEGF, IL-6, and TNF-α in gastric tissue. This experiment used primer sequences provided in Table 2.

### 3.9. Western Blot Analysis

Gastric tissues from each group were weighed, frozen, and ground into a fine powder using liquid nitrogen. RIPA lysate and 1/100 PMSF were added separately, and the samples were incubated on ice for 2 h. Supernatants were collected after centrifuging the samples for 10 min at 4 °C at 12,000 rpm. Following the determination of protein concentration using the BCA method, loaded samples were electrophoresed on SDS-PAGE, transferred onto PVDF membranes, and subsequently blocked in 5% BSA at room temperature for 1 h. Primary antibodies (diluted as follows: 1:2000, NF-κBp65; 1:1000, P-NF-κBp65; 1:1000, FoxO3a; 1:1000, Bim; 1:20,000, GAPDH) were added and incubated with the membrane at 4 °C overnight. The membrane, incubated with primary antibodies, was washed with TBST five times, each time for 10 min. Subsequently, the corresponding secondary antibodies (anti-rabbit and anti-mouse, both at a dilution of 1:5000) were added and incubated with the membrane at 37 °C for 1 h. The membrane was again washed with TBST five times, each time for 10 min. An appropriate amount of luminescent liquid was applied to the PVDF membrane until it was fully covered, and images were captured using an ECL luminometer (ChemiScope 5300 Pro, Shanghai, China). A Gray value analysis was performed on the target protein bands using ImageJ 1.50e software, and the expression levels of NF-κBp65, P-NF-κBp65, FoxO3a, and Bim were determined.

### 3.10. Data Analysis

In this study, SPSS 17.0 (SPSS Inc., Chicago, IL, USA) and GraphPad Prism 9 (GraphPad Software, San Diego, CA, USA) were used for the statistical analysis and data visualization. A one-way ANOVA analysis was applied to compare data with normal distribution and homogeneity of variance. If significant differences were observed (*p* < 0.05), Dunnett’s *t*-test, an LSD analysis, or Bonferroni’s *t*-test were used for between-group comparisons. The Kruskal–Wallis H Test analysis was employed for data that did not follow a normal distribution or exhibited variance irregularities, and Dunnett’s T3 test was utilized for between-group comparisons when significant differences were detected (*p* < 0.05). All results were expressed as the mean ± standard deviation, and significance was defined as *p* < 0.05 or *p* < 0.01.

## 4. Discussion

Damage to and self-repair of the gastric mucosa play integral roles in the pathogenesis of gastric ulcers. Various factors, such as active oxygen, alcohol consumption, smoking, Helicobacter pylori infection, and frequent use of non-steroidal anti-inflammatory drugs, are known to affect the gastric mucosa, causing mucosal damage [10,11,15,36]. The self-repair process following gastric mucosal injury is closely linked to factors like gastric mucus secretion, the mucus–bicarbonate barrier, cell regeneration, antioxidant levels, nitric oxide, and prostaglandins. When the invasive factors are intensified, the reparative mechanisms weaken, disrupting the balance between them and leading to the onset or worsening of GU [10,37]. Therefore, the rational use of drugs to reduce invasive factors and enhance the self-repair function of the gastric mucosa is key to preventing and treating GU.

In this study, a polysaccharide named DOP was successfully extracted and isolated from fresh stems of *D. officinale*, and a water extraction and alcohol precipitation method was used. Various chemical analysis methods were employed to determine the physical and chemical properties and structural characteristics of DOP. This study evaluated the biological activity of DOP in the context of GU induced by acetic acid in animals, shedding light on its molecular mechanisms. The experimental results demonstrated that DOP, a polysaccharide derived from *D. officinale*, possessed anti-gastric-ulcer properties. The molecular weight range of DOP was 2.27 × 10^6^ KDa, 3.7 × 10^2^ KDa, and 0.46 KDa and DOP was found to consist of mannose and glucose. Furthermore, DOP exhibited characteristic infrared absorption peaks of polysaccharides. Stability tests indicated that the structure of DOP remained stable below 239 °C. The surface of DOP molecules displayed numerous closely linked particles of different sizes that formed a columnar structure. Animal experiments established that DOP effectively ameliorated GU, attributed to its role in promoting gastric mucosal repair, inhibiting mucosal cell apoptosis, controlling oxidative stress and inflammatory factors in tissues and serum, and modulating the transcription of genes such as EGF, VEGF, IL-6, and TNF-α.

The rat model of acetic-acid-induced GU used in this study closely resembles chronic GU in humans [38]. During the establishment of the GU model, acetic acid inflicted damage on gastric mucosal tissues and cells, leading to inflammation. As the number of damaged cells increased, more immune cells were stimulated to release inflammatory factors, resulting in chronic inflammation that expedited ulcer formation [39,40,41]. In addition, excessive reactive oxygen species (ROS) played a significant role in accelerating GU formation. The elevated levels of MDA in gastric mucosa and serum indicated increased ROS, attributed to two primary reasons: First, the structure of gastric mucosa cells is disrupted, leading to mitochondrial damage and the release of more ROS [42]. On the other hand, macrophages can also release ROS. When acetic acid damages gastric mucosal cells, macrophages rapidly respond and migrate to the affected area, releasing pro-inflammatory factors like IL-1β, IL-6, and TNF-α, resulting in inflammation [43] and increased ROS generation. Antioxidants such as SOD, GSH, and GSH-PX are capable of scavenging ROS. The experimental results revealed that the serum levels of SOD, GSH, and GSH-PX in the model group were significantly lower than in the sham operation group, suggesting an imbalance between the oxidative and antioxidant systems in the gastric mucosa of rats in the model group. In this context, exacerbated inflammation and oxidative stress increased the apoptosis rate of gastric mucosa, disrupting the dynamic balance of cell proliferation and apoptosis and accelerating ulcer formation [44]. This highlights the critical relationship between ulcer healing and decreased apoptosis [45,46]. This point was confirmed by evaluating ulcer inhibition rates, TUNEL staining, and the Western blot analysis.

The healing of GU is a process of gastric gland remodeling, which will eventually lead to scar formation. This process is controlled by growth factors and cytokines, and the triggers activated by these growth factors are tissue damage and hypoxia [47]. EGF is crucial for cell growth, movement, and tissue repair, while VEGF is essential for blood vessel regeneration. When acetic acid stimulates the body, it triggers the activation of EGF and VEGF, leading to improved healing of GU. This study found that DOP groups did not increase the expression levels of EGF and VEGF in rat stomach tissue compared to the model group. This may be because the gastric ulcer healed after treatment in the DOP-200 and DOP-400 groups, leading to a decrease in EGF and VEGF levels. Overall, DOP was shown to promote gastric ulcer healing.

Plant polysaccharides are known for their potent antioxidant activity, primarily achieved by regulating the downstream antioxidant enzyme expression through the endogenous antioxidant stress pathway Nrf2/ARE. High antioxidant enzyme expression can effectively block the chain reaction of free radicals, reducing their production. Additionally, the inhibition of iNOS mRNA expression reduces NO production, thereby enhancing antioxidant capacity and mitigating oxidative stress damage [48]. The anti-inflammatory effect of plant polysaccharides is another prominent biological activity, which involves not only the direct downregulation of pro-inflammatory cytokine expression but also their interaction with antioxidant stress responses and the NF-κB cell signaling pathway [49]. Therefore, it can be inferred that the mechanism of DOP against acetic-acid-induced GU is multifaceted.

## 5. Conclusions

DOP ameliorates gastric ulcers by inhibiting the apoptosis of gastric mucosal cells, increasing antioxidant enzyme activity in gastric tissue and serum, promoting gastric mucosal repair, reducing the expression of pro-inflammatory factors, and downregulating the transcription of *IL-6* and *TNF-α* genes.

## Figures and Tables

**Table 1:** Monosaccharide Composition of DOP.

Monosaccharide Name	Peak Time of Standard (min)	Peak Time of DOP (min)	Peak Area	Content (%)	Specific Value
D-mannose	14.606	14.234	22,111,913	7.46	4.71
rhamnose	20.401	-	-	-	-
D-glucuronic acid	21.847	-	-	-	-
D-galacturonic acid	24.899	-	-	-	-
D-glucose	31.107	30.417	3,389,729	1.58	1.00
galactose	35.458	-	-	-	-
D-xylose	37.682	-	-	-	-
L-(+)-arabinose	39.080	-	-	-	-
fucose	46.170	-	-	-	-

**Table 2:** Primer sequences for real-time PCR.

Gene	Forward Primer	Reverse Primer
EGF	ACGGAGGGAGGCTACAAC	GGTCCACGGATTCAACAT
VEGF	AATCCTGGAGCGTTCACT	TCACCGCCTTGGCTTGTC
IL-6	TGCCTTCTTGGGACTGATG	TACTGGTCTGTTGTGGGTG
TNF-α	CGTAGCAAACCACCAAGCG	GGTATGAAATGGCAAATCG
GAPDH	ACAGCAACAGGGTGGTGGAC	TTTGAGGGTGCAGCGAACTT

## Data Availability

The original contributions presented in the study are included in the article, further inquiries can be directed to the corresponding author.
